# Single-Electrode Deep Brain Stimulation of Bilateral Posterolateral Globus Pallidus Internus in Patients With Medically Resistant Lesch-Nyhan Syndrome

**DOI:** 10.7759/cureus.37070

**Published:** 2023-04-03

**Authors:** Eliza Baird-Daniel, Adam Glaser, Scott Boop, Sharon Durfy, Jason S Hauptman

**Affiliations:** 1 Neurological Surgery, University of Washington, Seattle, USA; 2 Neurosurgery, Brigham and Women's Hospital, Boston, USA

**Keywords:** case report, neurosurgery, pediatric surgery, deep brain stimulation, lesch-nyhan syndrome

## Abstract

Deep brain stimulation (DBS) targeting various locations within the globus pallidus internus (GPi) is emerging as a therapeutic option for patients with medically resistant Lesch-Nyhan syndrome. We report our institutional experience with single-electrode DBS in the bilateral posterolateral GPi as an effective method for reduction of both dystonia and self-injurious behavior. Two pediatric patients aged six and 14 years underwent implantation of bilateral singular DBS leads in the posterolateral GPi and were followed postoperatively through the programming process and symptomatic improvements. Caregivers reported that after DBS in the posterolateral GPi, these patients experienced decreased self-mutilation behavior and decreased dystonia.

## Introduction

Lesch-Nyhan syndrome (LNS) is a rare X-linked inborn error of purine metabolism and is caused by the absence or partial absence of the enzyme hypoxanthine-guanine phosphoribosyltransferase (HPRT). First described by William Nyhan and Michael Lesch in 1964, the incidence is estimated to be one in 380,000 live births [1,2]. Complete absence of HPRT is characterized by hyperuricemia, urate crystal nephropathy, cognitive delay, dystonia, spasticity, and choreoathetosis, and the hallmark symptom of self-mutilation leads to considerable medical and social morbidity. Motor symptoms and cognitive delay typically present before one year of age, while onset of self-mutilation typically occurs between one and six years of age. Partial absence of HPRT is also possible. To date, 127 genetic variants of HPRT have been characterized [3]. Early diagnosis and treatment are critical, and while sudden death has been reported, with appropriate treatment, life expectancy can be into the second and third decades of life [2].

Few therapeutic options are available for LNS. Pharmacologic approaches include treating with allopurinol, oral and intrathecal baclofen, diazepam, clomipramine, risperidone, cyamemazine, L-5-hydroxytryptophan, L-dopa, botulinum toxin injections, and dopamine antagonist ecopipam [4-8]. The return to normal uric acid levels via administration of allopurinol minimizes nephropathy but does not address neurobehavioral or motor symptoms [9]. Baclofen and diazepam target motor symptoms of dystonia and spasticity with mixed results [10]. Practical treatments for neurobehavioral symptoms have included teeth extraction, physical restraints, botulinum toxin injections, and limb attachment [11]. While these interventions offer self-protection, they can be disruptive to daily functioning, may offer only temporary relief, and do not target the underlying pathology of LNS.

Interest in using deep brain stimulation (DBS) of the globus pallidus internus (GPi) for LNS stems from the efficacy of DBS of the GPi for treatment of both adult and pediatric dystonia [12]. To date, there have only been 14 published cases of DBS for LNS, and results have been variable [11]. Here we report our single-center experience with two patients receiving bilateral single-electrode DBS of the posterior GPi via robotic-assisted stereotaxy (ROSA). We report their follow-up, including postoperative adverse events, changes in disease management and medication changes, and caregiver assessment of patient outcome.

## Case presentation

A retrospective review of the electronic medical record was performed to identify LNS patients who received DBS of the posterior GPi at Seattle Children’s Hospital, Seattle, WA, USA. Informed consent was not requested or required by our Institutional Review Board for this case report.

The surgical target for both patients was the posterolateral aspect of bilateral GPi determined via Schaltenbrand atlas coordinates. Preoperative magnetic resonance imaging (MRI) was loaded onto the ROSA planning platform, and plans were determined using fast gray matter acquisition T1 inversion recovery (FGATIR) MRI sequences. Each patient underwent general anesthesia for implantation of bilateral single leads to the posterolateral GPi. A pre-planned trajectory via a coronal approach was used, and ROSA-guided stereotaxy with preoperative MRI, O-arm technology, and five bony fiducials ensured accuracy of electrode implantation. The internal pulse generator was placed during a second phase of the same surgery in the left anterior upper chest and was connected to the lead extensions via shunt passer tunneling. Postoperative computed tomography scans demonstrated appropriate placement of leads and did not demonstrate immediate complications.

Case 1

**Figure 1:**
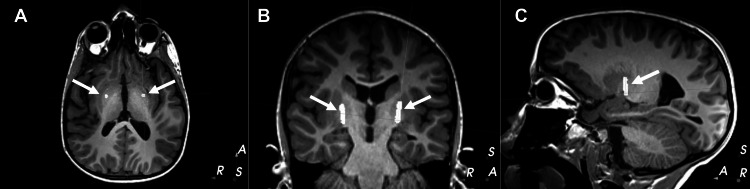
Depiction of bilateral single-electrode implantation sites generated from mapping of stereotactic target onto MRI for patient 1. (A) axial, (B) coronal, and (C) sagittal planes. Implantation sites are indicated by white arrows. MRI, magnetic resonance imaging

A six-year-old male, who lived at an inpatient assisted living facility, presented with symptoms of hyperuricemia, developmental delay, and worsening self-injurious behavior. He was non-verbal at baseline but demonstrated understanding, and ate a pureed and thick liquid diet due to dysphagia. His family sought surgical treatment for his medically refractory LNS following worsening of self-injurious behavior. Surgical implantation was performed in accord with preoperative MRI planning (Figure 1). No immediate postoperative complications occurred, and the patient’s last follow-up was at 21 months after surgery.

The patient underwent device programing at Oregon Health and Sciences University (Portland, OR, USA). Initial programming was monopolar with use of the third contact bilaterally. Pulse width was initially 200 ms with a frequency of 130 Hz. Transient decreased agitation was observed with titration of amplitude to 1.2 mAmps; however, further up-titration was not tolerated due to increased agitation. Final amplitude settings were pulse width of 450 ms and amplitude of 0.4 mAmps.

The patient’s caregivers report an estimated 75% reduction in the patient’s self-injurious behavior and in dystonia interfering with daily care. Additionally, caregivers noted increased patient “comfort” and more “purposeful movement.” Preoperatively, the patient was being evaluated for tooth extraction with the goal of decreased self-injury. Postoperatively, this procedure has been deemed unnecessary due to his symptomatic improvements. This patient continues to live at an inpatient assisted living facility.

Case 2

**Figure 2:**
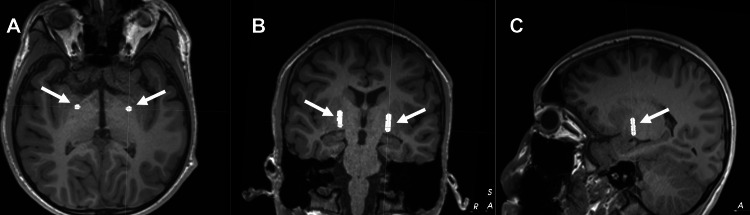
Depiction of bilateral single-electrode implantation sites generated from mapping of stereotactic target onto MRI for patient 2. (A) axial, (B) coronal, and (C) sagittal planes. Implantation sites are indicated by white arrows. MRI, magnetic resonance imaging

A 14-year-old male presented primarily with symptoms of severe, constant dystonia of the extremities and trunk, occasional choreiform activity, and self-injurious behavior. He was verbally responsive but impaired due to buccal dystonia, and demonstrated good understanding. While previously able to ambulate with a walker, worsening of his lower extremity and buccal dystonia necessitated wheelchair use. He had a bite-block in place. His self-injurious behaviors included biting arms and fingers and head banging. He was receiving levodopa-carbidopa at the time of presentation and had previously trialed trihexyphenidyl without significant clinical benefit. He underwent surgery at 13 years and has been followed for 22 months with bilateral implantation of single electrodes at GPi (Figure 2).

The patient underwent initial programming at Harborview Medical Center (Seattle, WA, USA). Programming was monopolar. Pulse width was 200 ms with a frequency of 119 Hz bilaterally. Improvement of buccal dystonia and speech was noted with increasing amplitude; however, at 2.5 mA on the left and 3.0 mA on the right, the patient experienced a sensation of “shakiness” in his head and he became tearful. Final amplitude settings were 0.5-2.5 on the left and 0.5-3.0 on the right.

Postoperatively, the patient developed high impedances on electrode 7 and 8 bilaterally and underwent revision of the generator, with lead extension replacement. He then developed wound breakdown at the scalp requiring revision of the distal lead and chest pocket revision to improve skin coverage. Since this revision, his caregivers noted significant improvement in dystonia symptoms and estimated a 20% decrease in buccal and lower extremity dystonia. This has enabled decreased use of restraints in the lower extremities. His family also reported a decrease in impulsivity, allowing for significantly less stringent supervision in tasks, including diaper changes.

## Discussion

We describe the cases of two patients with LNS who were treated at our institution with DBS of the GPi, both of whom experienced benefit as reported by their caregivers, including decreased self-mutilating behaviors for one patient and decreased dystonia for the second patient.

DBS is becoming an increasingly utilized treatment for medically refractory disorders in adult populations, including Parkinson disease, essential tremor, obesity, and obsessive-compulsive disorder [13]. More recently, there has been further development of DBS in pediatric populations, with promising results, including in dystonia, Tourette syndrome, and epilepsy.

Our single-center experience with DBS for LNS demonstrates that single-electrode implantation in bilateral GPi may be an effective treatment option to achieve symptomatic improvement as assessed by primary caregivers. Our experience also underlines some of the challenges that arise in the neurosurgical care of the LNS patient population. This patient population is at an elevated risk of wound breakdown, given propensity for self-injury and compulsive behavior, as reflected in the postoperative course described in case 2. Our experience with surgical site complication is consistent with that previously reported by other groups, in which implant infection was the most frequently reported complication [14].

There are unique challenges associated with device programing in pediatric patients due to young age and high potential for communication difficulties, which make it complicated to assess patient discomfort. In addition, unlike patients who undergo DBS programing for more easily quantifiable metrics such as tremor, there are many variables contributing to behavioral symptoms in LNS, and real-time quantifiable assessment of mood and self-injurious behavior is difficult. Symptoms such as dystonia are more readily assessed and therefore may be more treatable at this stage. The agitation associated with higher amplitude experienced by our case 1 patient may be associated with preferential stimulation of limbic GPi as opposed to motor due to lead location. In addition to more classically described side effects of electrode displacement in GPi stimulation, these anatomic considerations must be taken into account when counseling patients and families on stimulation side effects due to limbic overstimulation. Identification of reliable neuronal biomarkers and application of adaptive DBS is likely to increase efficacy and efficiency of the programming process.

While much of the research regarding pathophysiology of LNS focuses on the phenomenon of dopaminergic downregulation, studies aimed at pharmacologically increasing dopaminergic activity with agents such as L-Dopa, used in Parkinson disease, have led to inconsistent and even paradoxical results [5,15]. It appears that dopaminergic dysregulation may be a more complex phenomenon involving aberrant connectivity within the basal ganglia, limbic, and motor circuits.

GPi is an output nucleus of the basal ganglia. Anatomic immunohistochemistry studies demonstrate three functional regions of the GPi (limbic, associative, sensorimotor), indicating diffuse connectivity with multiple circuits implicated in the LNS disease process [16,17]. Initial imaging and autopsy studies of LNS patients did not demonstrate obvious neuroanatomic abnormalities; however, a quantitative MRI analysis of seven LNS patients demonstrated a consistent loss of basal ganglia volume [18].

Recent case reports of DBS of the GPi have helped elucidate the poorly understood pathophysiology of LNS. The initial report of single-electrode DBS to the GPi by Taira et al. [19] demonstrated improved motor symptoms and self-mutilating behavior, thus solidifying the role of GPi in both motor and neurobehavioral symptoms. Cif et al. [20] implanted electrodes bilaterally, one in the anteroventral (limbic) and posterior (sensorimotor) aspects, respectively. Discontinuation of the anterior leads caused return of self-injurious behavior, while discontinuation of posterior leads caused return of the motor symptoms, thus demonstrating the distinct functional networks within the GPi causing separate sets of symptoms in LNS [20]. This report suggests that distinct anterior and posterior networks in the GPi are implicated in the neurobehavioral and motor symptoms of LNS, respectively.

This report has some limitations. This is a small retrospective case series with two patients. Surgeries were performed at a single institution. Outcome was assessed based on subjective caregiver report. Given the severity of disease in these two patients, subjective caregiver report was determined to be the most appropriate approach, and caregiver reports provided valuable information on patient symptoms and behavioral changes. In future, it will be important to utilize objective outcome measures whenever possible.

## Conclusions

Our single-center experience with bilateral implantation of single-electrode DBS in two patients with LNS shows promising results in reduction of self-mutilating behavior and dystonia as reported by primary caregivers. As described by previous accounts, surgical site infection remains a significant challenge in this patient population due to self-injurious behavior. Our experience suggests that bilateral single-electrode implantation may reduce possible hardware malfunction as compared to multiple bilateral electrodes while still providing symptomatic benefit.
